# The Disposal of Sewage in India

**Published:** 1899-11

**Authors:** 


### THE DISPOSAL OF SEWAGE IN INDIA.

An experiment has just been brought to a successful conclusion in the Presidency Gaol, Calcutta, which is likely to have an important bearing upon the future sanitation of India. It has been to try the efficacy of the new aseptic pit system for the disposal of sewage, which has been very favorably reported upon at home. The aseptic pit it uncommonly like the old unwholesome cess-pit. It is managed, however, on scientific principles, and therein lies all the difference. The cess-pit in the new system is a dark, underground chamber, with well-luted sides, in which is cultivated the aseptic germ, a microscopic organism which has only to be introduced and supplied with a properly regulated flow of sewage to convert the evil-smelling products of the drains into harmless fluid. The sewage is allowed to remain in the pit just long enough to allow the necessary chemical changes to take place. This done the product passes out through a rough filter of well-aërated slag, where it becomes thoroughly deodorized, eventually leaving the apparatus in the form of a limpid and harmless product.

Extraordinary as it may appear, this product can not only be used with advantage for enriching the soil for crops, but is so innocuous that goldfish have been raised in it successfully at home, while in the case of the Calcutta experiment the tank-fish placed in the receptacle at the end of the series are growing fat and thriving. Exact statistics of cost and requisite dimensions have yet to be worked out, but in the event of these proving as low as expected, a future of great utility awaits the system. In the case of Calcutta, for example, it would be easy to adapt it to the insanitary slums in the north, where the sewers have never completely penetrated. While mofussil stations, as yet innocent of sewers, will shortly be able to consider it as an alternative. One of its chief advantages is that it enables the sewage from a single house or group of houses to be disposed of independently upon the spot, thus saving the heavy cost of sewage channels.—*Health*, Sept., 1899.

